# Immediate placement of intrauterine device after second‐trimester medical abortion—Secondary outcomes with one‐year follow‐up

**DOI:** 10.1111/aogs.70259

**Published:** 2026-06-10

**Authors:** Sara Hogmark, Caroline Westermark, Niklas Envall, Kristina Gemzell‐Danielsson, Helena Kopp Kallner

**Affiliations:** ^1^ Department of Clinical Sciences Danderyd Hospital Karolinska Institutet Stockholm Sweden; ^2^ Center for Clinical Research Dalarna, Uppsala University Falun Sweden; ^3^ Department of Obstetrics and Gynecology Danderyd Hospital Stockholm Sweden; ^4^ Department of Women's and Children's Health Karolinska Institutet Stockholm Sweden; ^5^ School of Health and Welfare, Dalarna University Falun Sweden; ^6^ Division of Gynecology and Reproductive Medicine, WHO‐Centre Karolinska University Hospital Stockholm Sweden

**Keywords:** contraception, family planning services, induced abortion, intrauterine device expulsion, intrauterine devices, long‐acting reversible contraception, randomized controlled trial

## Abstract

**Introduction:**

Intrauterine devices (IUDs) effectively prevent unwanted pregnancies. Little is known about long‐term outcomes of women choosing an IUD after second‐trimester medical abortion.

**Material and Methods:**

We performed a 12‐month follow‐up of a multicenter randomized controlled trial comparing IUD placement within 48 h (intervention) with placement at 2–4 weeks (control) after medical abortion with 85 to 153 days' gestation. Secondary outcomes such as proportions of long‐term IUD use, subsequent pregnancies and abortions, and participant satisfaction are presented (ClinicalTrials.gov NCT03603145).

**Results:**

A total of 179 women were included. The 12‐month follow‐up rate was 71.1% (64/90) in the intervention group and 73.0% (65/89) in the control group. Fewer women in the intervention group were using an IUD after 12 months: 51.6% (33/64) vs. 69.2% (45/65, *p* = 0.04). The total expulsion rate over 12 months was 36.4% (24/66) in the intervention group compared to 3.1% (2/65) in the control group (*p* < 0.001), with only two IUDs being expelled between 6 and 12 months, both in the intervention group. The most common contraceptive method at 12 months was any hormonal IUD at 54.3% (70/129). Among IUD users at 12 months, the overall satisfaction with their contraceptive method was high, at 88.3% (68/77). In the intervention group, 26.2% (16/61) had at least one subsequent pregnancy within 12 months compared to 13.6% (9/66, *p* = 0.08) in the control group. We found no significant difference in the number of subsequent abortions; 13.1% (8/61) in the intervention group and 6.1% (4/66) in the control group reported at least one subsequent abortion (*p* = 0.23).

**Conclusions:**

Immediate IUD placement after second‐trimester medical abortion did not lead to a higher proportion of IUD use at 12 months compared to delayed placement. Rather, immediate IUD placement was associated with a higher incidence of expulsion and lower use; however, no significant increase in subsequent pregnancies or abortions was observed. High satisfaction levels with IUD use were reported in both groups, but women with immediate placement were less likely to recommend a friend to use an IUD post abortion.

## Graphical abstract

Immediate intrauterine device placement after second‐trimester medical abortion was associated with a higher incidence of expulsion, lower use of IUD at 12 months, and lower acceptance compared with delayed placement. However, no significant increase in subsequent pregnancies or abortions was observed at the 12 month follow‐up.

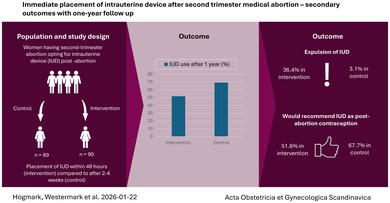

**Abbreviations**
Cu‐IUD: Copper intrauterine device
H‐IUD: Hormonal intrauterine device
IUD: Intrauterine device

**Key message**

Immediate placement of an intrauterine device (IUD) following second‐trimester medical abortion is associated with a higher risk of IUD expulsion and does not result in increased IUD utilization or improved acceptability at 1 year post abortion, compared to delayed placement.

## 1 INTRODUCTION

Starting contraception after any abortion prevents new unwanted pregnancies, with long‐acting reversible contraception being associated with fewer subsequent abortions than short‐acting methods.^1^, ^2^, ^3^, ^4^ For intrauterine contraception, there have been few studies into the optimal timing of placement after second‐trimester medical abortion, and none have been adequately powered.^5^, ^6^ Yet, the World Health Organization states that benefits of immediate placement of intrauterine devices (IUDs) after any abortion in the second trimester generally outweigh risks.^7^ When determining the optimal timing for IUD placement, placement rates and long‐term expulsion and removal rates need to be considered.

The parent trial of this study was a randomized controlled trial of IUD placement within 48 hours or at 2–4 weeks after the abortion. In that trial, we could not show superiority with immediate placement in terms of IUD use at 6 months post abortion (primary outcome).^8^

The objective of the current study was to analyze secondary outcome data from this trial such as type of IUD chosen and reasons for this choice, continuation and discontinuation, and satisfaction of contraceptive methods at 12 months post‐abortion.

## 2 MATERIAL AND METHODS

The complete study design has been previously described.^8^, ^9^ In short, women ≥18 years having medical abortion at 85–153 days of gestation and planning to use an IUD for post‐abortion contraception were randomized to placement within 48 hours (intervention) or at 2–4 weeks post‐abortion (control). Estimations on sample size and power calculations are further specified in the previous trial.^8^ We aimed to include 120 participants in each group. The trial was prematurely stopped due to a predefined stopping criterion of expulsion >20% in the intervention group.

In this multicenter trial, medical abortions were performed in accordance with the World Health Organization guidelines.^7^ Participants chose their preferred type of IUD: levonorgestrel‐IUD 52, 19.5, or 13.5 mg (collectively called hormonal IUDs, H‐IUD), or copper IUD (NovaT 380™), manufactured by Bayer GmBH, Leverkusen, Germany. IUDs were supplied free of charge, in accordance with the study requirements set by the Medical Products Agency and were placed at the abortion clinics. Replacement IUDs and visits for this were not part of the study protocol but were provided within the regular health system.

Data were collected through follow‐up questionnaires sent to participants per e‐mail at 3, 6, and 12 months, or by telephone in case of non‐reply. No clinical follow‐up visits were scheduled. If participants reported having attended a medical appointment, relevant information was obtained from the electronic health record in accordance with the informed consent. The analysis of the primary outcome, IUD use at 6 months, has been published.^8^ This publication presents secondary outcomes including reasons for choosing a specific IUD, contraceptive use, and satisfaction at 12 months post‐abortion, acceptability, and subsequent pregnancies and abortions.

We dichotomized satisfaction as satisfied (very satisfied/satisfied/neither) or dissatisfied (dissatisfied, very dissatisfied). For acceptability, participants were asked whether they would recommend a friend to choose an IUD as post‐abortion contraception (yes/no/do not know).

### 2.1 Statistical analyses

For statistical analysis, IBM SPSS Statistics for Windows version 29.0 and R for Windows version 4.2.2 were used. The analyses were conducted on the complete dataset, using only observed outcomes and without any imputation of missing data. Thus, the denominator for each result may vary depending on the number of respondents to a given question and on the availability of corresponding health record data.

Non‐normally distributed continuous variables are reported as medians with interquartile range (25th–75th percentile) along with minimum and maximum values; Mann–Whitney *U*‐test was used to explore group differences. Dichotomous variables are presented as proportions, and group differences were assessed using the Chi‐squared, Fisher's exact, or Fisher–Freeman–Halton test. Results were considered significant if the two‐sided *p*‐value was <0.05.

Predictors of continued IUD use at 12 months were estimated using a modified Poisson regression model adjusted for robust error variance.^10^ Covariates were selected based on existing evidence of presumed clinical relevance: attendance to IUD placement visit (yes/no), age (≤ or >30 years), level of education (≤12 years/≥13 years), parity (nulliparous/parous), history of previous abortion (yes/no), and whether participants reported any of the following main reasons for choosing a specific IUD: positive health effects, effectiveness, low risk of side effects, low dose or no hormones, recommendation from healthcare provider, or belief that IUDs are comfortable or easy to use. Results are presented as risk ratios (RR) with 95% confidence intervals (CI).

## 3 RESULTS

We randomized 179 women to intervention (*n* = 90) or control (*n* = 89). Baseline characteristics of women, primary and secondary outcomes at 6 months follow‐up have been previously described.^8^ In short, the median participant age was 28 years (IQR 24–34; range 18–44), with 60.4% (99/165) being parous, and 55.8% (92/165) had a history of abortion. The median gestational length at abortion was 99 days (IQR 91.5–111; range 72–153). Attendance rate at the placement visit was 89.6% (69/77) and 71.8% (56/78) in the intervention and control groups, respectively (*p* = 0.008).^8^ A total of 85.2% (69/81) and 72.2% (58/79) in the intervention and control groups, respectively, received an IUD post‐abortion, also including IUD placement at surgical intervention for retention of products of conception at any time (*p* = 0.07).

### 3.1 Reasons for choosing IUD for contraception

Overall, high contraceptive effectiveness was the main reason for choosing an IUD, reported by 43.1% (66/153), followed by finding it comfortable/easy to use at 15.7% (24/153) (Table 1). The most common reason for choosing the Cu‐IUD was low hormone dose/no hormones 40% (6/15). The main secondary reason for choosing an IUD was comfortability 60.5% (75/124), followed by a wish to reduce or avoid bleeding 35.5% (44/124) and it being a long‐acting method 26.6% (33/124) (Table 2).

**TABLE 1:** Main reasons for choosing a specific type of intrauterine device after second‐trimester medical abortion (*n* = 179).

Reason^a^, ^b^	Type of IUD chosen				
	H‐IUD 52 mg, *n* = 102	H‐IUD 19.5 mg, *n* = 43	H‐IUD 13.5 mg, *n* = 4	Cu‐IUD, *n* = 15	Total, *n* = 164^d^
	*n* (%)	*n* (%)	*n* (%)	*n* (%)	*n* (%)
Effective	48 (50.5)	14 (35.0)	0 (0.0)	4 (26.7)	66 (43.1)
Comfortable/Easy to use, no need to think about it	11 (11.6)	9 (22.5)	1 (33.3)	3 (20.0)	24 (15.7)
Low hormone dose/no hormones	5 (5.3)	6 (15.0)	0 (0.0)	6 (40.0)	17 (11.1)
Recommended by HCP	11 (11.6)	6 (15.0)	0 (0.0)	0 (0.0)	17 (11.1)
Wish to reduce or avoid bleeding	11 (11.6)	3 (7.5)	0 (0.0)	0 (0.0)	14 (9.2)
Long‐acting	3 (3.2)	1 (2.5)	0 (0.0)	1 (6.7)	5 (3.3)
Other reason	2^e^ (2.1)	0 (0.0)	2^f^ (66.7)	0 (0.0)	4 (2.6)
Eases dysmenorrhea	3 (3.2)	0 (0.0)	0 (0.0)	0 (0.0)	3 (2.0)
Low risk side effects	0 (0.0)	1 (2.5)	0 (0.0)	1 (6.7)	2 (1.3)
Recommended by relative/friend	1 (1.1)	0 (0.0)	0 (0.0)	0 (0.0)	1 (0.7)
Missing^c^	7 (7.4)	3 (7.5)	1 (33.3)	0 (0.0)	11 (7.2)

Abbreviations: Cu, copper; HCP, healthcare provider; H‐IUD, levonorgestrel hormonal intrauterine device.

^a^ Proportions are calculated based on participants who responded to the question; missing responses are excluded.

^b^ Two main reasons—discrete and cost—were not reported by any of the participants as main reason.

^c^ Missing proportion represents those not answering the question or giving more than one main reason for choosing type of IUD.

^d^ Fifteen participants were excluded or withdrew their participation, hence, total *n* = 164.

^e^ Two participants reported choosing H‐IUD 52 mg for other reasons, one due to previous good experience and the other reported not being able to use any other contraceptive method.

^f^ Two participants choosing H‐IUD 13.5 mg reported other reasons, one because she had had it before and the other because the IUD was smaller than the H‐IUD 52 mg which she had before.

**TABLE 2:** Secondary reasons for selecting a certain type of intrauterine device after second‐trimester medical abortion (*n* = 179).

Reason^a^, ^b^	Type of IUD chosen				
	H‐IUD 52 mg, *n* = 102	H‐IUD 19.5 mg, *n* = 43	H‐IUD 13.5 mg, *n* = 4	Cu‐IUD, *n* = 15	Total, *n* = 164^d^
	*n* (%)	*n* (%)	*n* (%)	*n* (%)	*n* (%)
Comfortable/Easy to use, no need to think about it	47 (61.8)	21 (63.6)	1 (33.3)	6 (50.0)	75 (60.5)
Wish to reduce or avoid bleeding	34 (44.7)	9 (27.3)	1 (33.3)	0 (0.0)	44 (35.5)
Long‐acting	22 (28.9)	7 (21.2)	1 (33.3)	3 (25.0)	33 (26.6)
Recommended by HCP	19 (25.0)	8 (24.2)	1 (33.3)	3 (25.0)	31 (25.0)
Effective	14 (18.4)	8 (24.2)	1 (33.3)	4 (33.3)	27 (21.8)
Low hormone dose/no hormones	14 (18.4)	7 (21.2)	1 (33.3)	4 (33.3)	26 (21.0)
Eases dysmenorrhea	10 (13.2)	5 (15.2)	0 (0.0)	0 (0.0)	15 (12.1)
Recommended by relative/friend	5 (6.6)	4 (12.1)	0 (0.0)	0 (0.0)	9 (7.3)
Low risk side effects	4 (5.3)	2 (6.1)	0 (0.0)	2 (16.7)	8 (6.5)
Cost	4 (5.3)	3 (9.1)	0 (0.0)	0 (0.0)	7 (5.6)
Discrete	1 (1.3)	2 (6.1)	0 (0.0)	2 (16.7)	5 (4.0)
Other reason	2^e^ (26.3)	0 (0.0)	0 (0.0)	0 (0.0)	2 (4.0)
Missing^c^	26 (34.2)	10 (30.3)	1 (33.3)	3 (25.0)	40 (32.3)

Abbreviations: Cu, copper; HCP, healthcare provider; H‐IUD, levonorgestrel hormonal intrauterine device.

^a^ Proportions are calculated based on participants who responded to the question; missing responses are excluded.

^b^ Two main reasons—discrete and cost—were not reported by any of the participants as main reason.

^c^ Missing proportion represents those not answering the question.

^d^ Fifteen participants were excluded or withdrew their participation, hence, total *n* = 164.

^e^ Two participants reported other reasons for choosing IUD 52 mg; one reported H‐IUD being the only contraceptive method she had not used and the other one did not specify any reason.

### 3.2 Follow‐up at 12 months

Follow‐up rates at 12 months were 71.1% (64/90) and 73.0% (65/89) in the intervention and control groups, respectively (*p =* 0.77). The total loss to follow‐up rate was 27.9% (50/179), whereof 20 withdrew consent within 3 months, and 30 were not reached. A participant flowchart up to the 6‐month follow‐up has been previously published.^8^ A complementary flowchart, detailing the 6–12‐month follow‐up, is provided in Figure S1.

### 3.3 Use of contraceptive methods at 12 months post abortion

Among participants who answered the 12‐month questionnaire, IUD use at 12 months after the index abortion was 51.6% (33/64) in the intervention and 69.2% (45/65) in the control group (*p* = 0.04). In a secondary analysis of participants who had an IUD placed and who completed the 12‐month questionnaire, 54.4% (31/57) in the intervention group and 81.8% (45/55) in the control group were still using an IUD at 12 months after the index abortion (*p* = 0.002) with 36.8% (21/57) in the intervention group and 74.5% (41/55) in the control group (*p* < 0.001) using the same IUD as placed after the index abortion. The only significant predictor of IUD use after 12 months was attendance to the initial post‐abortion placement visit (OR 1.76, 95% CI 1.13–2.74, *p =* 0.01). See Table S1.

Overall, the most common contraceptive method at 12 months was any H‐IUD at 54.3% (70/129), followed by Cu‐IUD, 6.2% (8/129), and male condom at 6.2% (8/129). In the intervention group, 31.3% (20/64) reported using no contraceptive at 12 months compared to 20.0% (13/65) in the control group (*p* = 0.14). Overall satisfaction with contraceptive methods used at 12 months post‐abortion did not differ between groups and is presented in Table 3 (*p* = 0.20). Among IUD users, 88.3% (68/77) reported satisfaction, with no difference between groups (*p* = 0.48). Due to low numbers of users of other methods or no contraception, no further in‐between comparisons were made.

**TABLE 3:** Contraceptive methods used immediately and 12 months following second‐trimester medical abortion, and participant reported satisfaction at 12 months (full sample, *n* = 179).

Method	Immediately after abortion (*n* = 127)^a^		12 months after abortion (*n* = 129)^b^		Satisfied^c^, *n*/*n* (%)
	Intervention	Control	Intervention	Control	
IUD^d^	69	58	33	45	68/77 (88.3)
H‐IUD	62	54	28	42	62/69 (89.9)
Cu‐IUD	7	3	5	3	6/8 (75.0)
Condom	N/A	N/A	3	5	7/7 (100.0)
COC	N/A	N/A	3	0	3/3 (100.0)
Implant	N/A	N/A	1	1	1/2 (50.0)
POP	N/A	N/A	2	0	2/2 (100.0)
Patch	N/A	N/A	2	0	2/2 (100.0)
Other non‐hormonal contraception	N/A	N/A	0	1^e^	1/1 (100.0)
No method	N/A	N/A	20	13	24/29 (82.8)
Total	69	58	64	65	108/123 (87.8)

Abbreviations: COC, combined oral contraceptive pills; Cu, copper; H‐IUD, levonorgestrel hormonal intrauterine device; IUD, Intrauterine device; POP, Progestogen‐only pills.

^a^ A total of 19 participants withdrew their consent or were excluded prior to the placement visit. An additional 33 participants did not have an IUD placed following the index abortion.

^b^ At the 12‐month follow‐up, a total of 20 participants withdrew their consent or were excluded. An additional 30 participants did not respond to the question.

^c^ Six participants reported using contraception at 12 months, but did not report satisfaction. Among them, four reported no contraceptive use, one reported using a condom, and one reported using an H‐IUD. Thus, discrepancy is seen in the number of participants when comparing contraceptive method data with satisfaction responses in the table.

^d^ Any type of IUD, including both hormonal and copper IUD.

^e^ One participant in the control group reported using a contraceptive app.

### 3.4 IUD expulsion and discontinuation

At 6 months, 30.1% (22/73) of IUDs had been expelled in the intervention group compared to 2.9% (2/70) in the control group (*p* < 0.001).^8^ Between six and 12 months post abortion, two additional IUD expulsions were reported in the intervention group. One was partial with the IUD visible in the cervix; she reported using an H‐IUD at 12 months. The second was a complete expulsion detected due to heavy menstrual bleeding; she reported no contraceptive use at 12 months. Both became pregnant and had new abortions. Thus, among participants with data available at 12 months, IUD expulsion within the first year post abortion was 36.4% (24/66) in the intervention group compared to 3.1% (2/65) in the control group (*p* < 0.001). Eight of 24 expelled IUDs in the intervention group and both in the control group were replaced. Of the remainder, six reported no contraceptive use, three used combined pills, two progestogen‐only pills, one the patch, and one condom only at 12 months. In a secondary analysis including only those who had an IUD placed post‐abortion and completed the 12‐month questionnaire, 40.7% (24/59) in the intervention and 3.6% (2/55) in the control group had expulsions (*p* < 0.001) whereof 16/26 were complete, five were partial and the degree of expulsion was unknown for five participants. Expulsions by IUD type and group are detailed in Table 4. There were no differences in the proportion of expelled IUDs by type (hormonal or copper).

**TABLE 4:** Intrauterine device (IUD) expulsion per IUD type within 12 months of second‐trimester medical abortion with post‐abortion IUD placement (*n* = 127).^a^

IUD type	Intervention (*n* = 69)	Control (*n* = 58)
	Expulsions, *n*/*n* (%)	Expulsions, *n*/*n* (%)
H‐IUD	22/62 (33.3)	2/55 (3.6)
H‐IUD 52 mg	15/38 (36.6)	2/41 (4.9)
H‐IUD 19.5 mg	7/22 (31.8)	0/14 (0.0)
H‐IUD 13.5 mg	0/2 (0.0)	0/0 (0.0)
Cu‐IUD	2/7 (28.6)	0/3 (0.0)
Total	24/69 (34.8)	2/58 (3.4)
Missing or excluded	10^b^	3^c^

Abbreviations: Cu, copper; H‐IUD, levonorgestrel hormonal intrauterine device.

^a^ Including only women having an IUD placed.

^b^ In the intervention group, expulsion was unknown for 10 individuals, four with a H‐IUD 52 mg, five with a H‐IUD 19.5 mg, and one with a Cu‐IUD placed.

^c^ In the control group, expulsion was unknown for three individuals, two with a H‐IUD 52 mg and one with a H‐IUD 19.5 mg placed.

Discontinuation of IUD use at 12 months—that is, participants receiving an IUD after the index abortion with no IUD use at 12 months—differed significantly between the intervention and control groups, with 45.6% (26/57) and 18.2% (10/55) discontinuation in the intervention and control group, respectively (*p* = 0.002). The most common reasons were IUD expulsion, followed by bleeding problems and a wish to become pregnant. See Table S2 reporting reasons other than expulsion for IUD discontinuation.

### 3.5 Acceptability

A total of 51.6% (33/64) in the intervention group would recommend a friend to use an IUD for post‐abortion contraception, while 26.6% (17/64) were indecisive and 21.9% (14/64) would not recommend it. This differed significantly from the control group where the corresponding numbers were 67.7% (44/65), 26.2% (17/65), and 6.2% (4/65) (*p* = 0.027). In both groups, eight women indicated that they would still recommend an IUD as a post‐abortion contraceptive method, despite no longer using one themselves.

### 3.6 Pregnancies at 12 months post abortion

In the intervention group, 26.2% (16/61) compared to 13.6% (9/66) in the control group had become pregnant at least once (*p* = 0.08). Of these, 15 women had a new pregnancy in the first 6 months after the index abortion and 10 between six to 12 months post‐abortion. Of all new pregnancies, 36% (9/25) were reported as intended, six in the intervention group and three in the control group. In the intervention group, 56.3% (9/16) of those who became pregnant during the 12‐month follow‐up had experienced IUD expulsion compared to 11.1% (1/9) in the control group (*p =* 0.002). In the intervention group, 13.1% (8/61) reported at least one subsequent abortion compared to 6.1% (4/66) in the control group (*p* = 0.23). Participants in the intervention group having had unprotected sex without a wish to become pregnant were 21.9% (14/64) vs. 12.3% (8/65) in the control group (*p* = 0.15).

## 4 DISCUSSION

In our study, the higher proportion of women who received the IUD in the immediate group could not compensate for the higher expulsion rate in this group at the one‐year follow‐up. Still, most IUDs were not expelled. Most of the expulsions were complete, and very few additional expulsions occurred after 6 months post‐abortion. Satisfaction among those still using an IUD at 12 months post‐abortion was high in both groups. Acceptability of the IUD as post‐abortion contraception was higher among women receiving the IUD at 2–4 weeks post‐abortion compared to within 48 h. No significant differences were seen in subsequent pregnancies or abortions. Women who chose the copper IUD for post‐abortion contraception were less satisfied than those who chose the hormonal IUD. These results are similar to those from the CHOICE study.^11^ However, the number of women who chose the copper IUD in our study was few and the study is not powered to study these differences in acceptability.

Few studies have reported on long‐term retention of effective contraception after second‐trimester medical abortion. In contrast to our findings, a previous study by Korjamo et al. on one‐year follow‐up on IUD placement within 3 days compared with placement at 2–4 weeks after second‐trimester abortion showed higher proportions of continued IUD use with early placement compared to delayed placement.^6^ However, the study did not report a definitive continuation rate at 12 months. Instead, the authors presented two scenarios: a worst‐case scenario for those with IUD placed and verified at 12 months, and a best‐case scenario based on the proportion with IUD placed and verified or with unknown use at 12 months. In the immediate placement group, IUD use ranged from 55.6% to 88.9%, whereas in the delayed group it ranged from 25.0% to 60.7%.^6^ The authors acknowledged that the study was underpowered with only 57 participants.^6^ Thus, we present the first larger study on long‐term post‐abortion IUD use which likely makes the results more reliable. We also report on the use of other contraceptive methods after second‐trimester medical abortion.

Previous studies have shown that women having second‐trimester abortion are at greater risk of a new abortion at any gestational week compared to those having first trimester abortion, as well as having a new second‐trimester abortion which is associated with more complications compared to early abortion.^12^, ^13^ This is in accordance with our study, where a number of participants also experienced new pregnancies and unwanted pregnancies with subsequent abortions. Therefore, finding an evidence‐based method for provision of effective contraception to prevent a new unwanted pregnancy is important, especially for this group. With a 12‐month follow‐up rate at 72.1%, compared to 93.8% after first trimester medical abortion,^9^ our study confirms previous findings of higher gestational length at time of abortion being associated with higher risk for loss to follow‐up.^3^

It has previously been reported that 42% of women scheduled for IUD placement 2–4 weeks after abortion do not return for the booked appointment. Moreover, evidence suggests that the risk of a new abortion is highest among women failing to attend the placement visit.^14^, ^15^ Our high attendance for placement in the delayed group could possibly be explained by the IUDs being provided for free, which differs from clinical practice in Sweden. Free IUDs and replacement IUDs were also provided in the Korjamo trial.^6^ We also contacted women several times if they missed their placement visit. Furthermore, evidence suggests that when IUD placement is provided as part of the abortion service, more IUDs are placed in comparison to referral and provision in primary health care (91.2% vs. 24.1%).^16^

Thus, organization of health care services affects attendance to the placement visit‐ the only factor that is clearly shown to affect use of IUD at 12 months apart from timing of placement. The organization of health care services therefore clearly affects short‐ and long‐term continuation rates of effective contraception. Women participating in randomized controlled trials may also differ from the general population and have higher attendance rates per se.

It could be argued that second‐trimester medical abortion shares some features with vaginal deliveries, such as greater cervical dilation and larger uterine size, compared to early gestation abortion. Studies on immediate postpartum IUD placement report high continuation rates and satisfaction despite expulsion rates exceeding those after second‐trimester abortion.^17^, ^18^ In a Swedish randomized controlled trial, 44.2% experienced expulsion, yet 75% used an IUD at 12 months postpartum, and 83.7% would recommend the method.^17^ Similarly, a UK trial by Cooper et al., offering immediate postpartum placement, follow‐up, and free‐of‐charge replacement in case of expulsion, reported 79.6% continuation of IUD use and 98.3% recommendation rate despite a 60.8% expulsion rate.^18^ In contrast to these results, in our study, control group participants were more inclined to recommend IUD post‐abortion than participants in the intervention group (67.7% vs. 51.6%). Pre‐placement counseling on expulsion risk and offering a structured follow‐up procedure with free‐of‐charge replacement IUDs may increase replacement and IUD use after second‐trimester abortion, as seen with postpartum placement.^18^ Differences in acceptability may also reflect population differences between those undergoing second‐trimester abortion and those giving birth. There were no differences in the proportion of expelled IUDs by type (hormonal or copper); however, the study is severely underpowered to study any such differences.

In clinical practice, our results indicate that in a setting where attendance at the delayed IUD placement visit is high, immediate placement does not lead to higher proportions of IUD use after 1 year, nor to fewer additional pregnancies or abortions. Rather, results point in the opposite direction, with fewer IUD users in the immediate group at 12‐month follow‐up with a tendency toward more unprotected intercourse and pregnancies. These results can be explained by the high risk for IUD expulsion in participants receiving immediate placement. Clinically, it is important to note that results in the second trimester differ from those in the first trimester abortion, where no clear difference in expulsion is seen.^9^ Still, after a second‐trimester abortion, most IUDs placed within 48 h remain in place, and high satisfaction rates were found in users remaining at 12 months. Women having second‐trimester abortion could be considered a vulnerable group with high risk of a new unintended and unwanted pregnancy, and individual assessment for those considered to benefit from immediate placement can increase use at 12 months post abortion. Providing free IUDs as part of the abortion care, sending reminders, and offering free‐of‐charge replacements could increase placements and improve effective contraceptive use post‐abortion.

More studies are needed to investigate the optimal timing for IUD placement after second‐trimester abortion. Finding methods to reduce expulsions should be a high priority. Further, characterizing specific predictors for women who would benefit from immediate placement, regardless of expulsion risk, would improve individualized counseling. Methods to increase acceptability of immediate placement, such as free IUDs and free replacements, should be explored. Additionally, investigating the timing of IUD placement in relation to gestational week–from first trimester abortion to postpartum–would broaden knowledge on IUD placement from a wider perspective.

A major strength of this study is the randomized controlled trial design. Additionally, the absence of routine ultrasound use before IUD placement or extra in‐clinic follow‐up enhances generalizability and applicability in various settings. Another strength is the relatively high attendance to the placement visit in both groups, resulting in more reliable data on expulsion risk. We also performed a long‐term follow‐up of women who had an abortion, their contraceptive use, satisfaction, and subsequent pregnancies, while exploring pregnancy intentions. Due to high drop‐out rates and a premature stop in recruitment, fewer women were included, and the study is therefore underpowered. It was not powered for the detection of rare events. Furthermore, blinding was not deemed feasible.

## 5 CONCLUSION

IUD placement within 48 h of second‐trimester medical abortion did not lead to a higher proportion of long‐term IUD use compared to placement within 2–4 weeks. Rather, immediate IUD placement was associated with a higher incidence of expulsion and lower use; however, no significant increase in subsequent pregnancies or abortions was observed. High satisfaction levels with IUD use were reported in both groups, but women with immediate placement were less likely to recommend a friend to use an IUD post abortion. In selected cases, immediate placement can be preferred and performed with reassuring safety and acceptability. Further studies on the timing of IUD placement and strategies to reduce expulsion after second‐trimester abortion are needed to offer more effective and acceptable post‐abortion contraception.

## AUTHOR CONTRIBUTIONS

HKK and KG‐D planned and conceived the study. HKK, SH, and CW managed the trial and performed data analyses. All authors participated actively in interpretation of data and in writing the paper. SH and CW wrote the first draft and made equal contributions to the paper. All authors approved the submitted paper.

## FUNDING INFORMATION

Funding was provided by the Swedish Society of Medicine (SLS‐692651) and the Stockholm Region/Karolinska Institutet (ALF, LS‐2016‐1376, RS 2019‐1054, K 0138‐2015, LS 2018‐1257). The financial support was used to cover the cost of the hormonal intrauterine devices used in the study, salaries for participating researchers, and staff recruiting participants and supervising the study. The funding sources were not involved in study design, the collection, analysis, or interpretation of data, the writing of the report, or the decision to submit the article for publication.

## CONFLICT OF INTEREST STATEMENT

C.W. reports no conflict of interest. S.H. reports honoraria from Bayer AG and Gedeon Richter Nordics AB. N.E. has received personal fees from Organon and Bayer for educational activities and an honorarium from Medsphere Corp USA for expert opinions on long‐acting reversible contraception, outside the submitted work. K.G.D. reports honoraria from Bayer AG, Organon/MSD, Gedeon Richter, Mithra, Natural Cycles, MedinCell, Ferring, Norgine, Exelgyn, Exeltis, Cirqle, RemovAid, and Obseva. H.K.K. reports honoraria from: Bayer, Bionorica/Agnomed, Campus Pharma, Gedeon Richter, Exeltis, Natural Cycles, Mithra, Consilient Health, Ferring, Gesynta, MSD/Organon.

## ETHICS STATEMENT

The study was approved by the Regional Ethics Committee of Stockholm, with amendments for additional study centers (permit numbers 2016/1685‐31/1 [approved October 7, 2016]; 2018/48‐32 [January 12, 2018]; 2018‐962‐32 [May 4, 2018]; 2019‐03183 [June 20, 2019]; 2020‐02925 [July 3, 2020]; and 2021‐02625 [June 18, 2021]), and by the Swedish Medical Products Agency (EudraCT number 2018‐000287‐29). It was registered on July 27, 2018 in ClinicalTrials.gov (NCT03603145). The first participant was enrolled in January 2019 and the last participant in June 2022.

## Supporting information


**Figure S1.** Participant flowchart.

**Table S1.** Poisson regression analysis of correlations between baseline covariates and IUD use at 12 months after second‐trimester medical abortion (*n* = 153^a^).

**Table S2.** Reasons for intrauterine device (IUD) discontinuation, other than expulsion, among women having an IUD placed after second‐trimester medical abortion (*n* = 127).

## Data Availability

The data that support the findings of this study are available on request from the corresponding author. The data are not publicly available due to privacy or ethical restrictions.
