# Supplementary material for: Immediate placement of intrauterine device after second‐trimester medical abortion—Secondary outcomes with one‐year follow‐up
Source: Acta Obstet Gynecol Scand. 2026 Jun 10;105(9):1672–9. doi: 10.1111/aogs.70259 (PMC13394263; doi:10.1111/aogs.70259)
Supplement: Supplementary file 1 [file AOGS-105-1672-s001.docx]

**Supporting Information Figure S1 – Participant Flowchart**

## Enrollment

Assessed for eligibility (n=195)

Excluded (n=16)

Randomized (n=179)

## Allocation

Allocated to control (n=89)

Allocated to intervention (n=90)

Discontinued before IUD placement (n=10)

Withdrew consent (n=4)

Excluded (n=6)

Discontinued before IUD placement (n=9)

Withdrew consent (n=3)

Excluded (n=6)

## Study participants n=79

## Study participants n=81

IUD placed (n=69)^a^

IUD placed (n=58)^b^

Lost to follow-up between 6-12 months (n=2)

Could not be reached (n=3^e^)

Lost to follow-up within 6 months (n=14)

Could not be reached (n=13)

Withdrew consent (n=1)

## Follow-Up

## after 6 months^c^

## Analyzed at 6 months n=67

Lost to follow-up between 6-12 months (n=3)

Could not be reached (n=5^d^)

## Analyzed at 12 months n=64

## Follow-Up

## after 12 months

Analyzed (n=65)
Excluded due to exclusion criteria or withdrawal of consent (n=11)

Could not be reached (n=13)

Analyzed (n=64)
Excluded due to exclusion criteria or withdrawal of consent (n=10)

Could not be reached (n=16)

Lost to follow-up within 6 months (n=12)

Could not be reached (n=11)

Withdrew consent (n=1)

## Analyzed at 12 months n=65

## Analyzed at 6 months n=67

## Analysis of IUD use at 12 months^f^

^a^Including all participants who had IUD placed, including women who received an IUD outside the allocated time window, those who had an IUD placed at surgery, and those who had IUD placed at clinics other than the study sites.

^b^Including all participants who had IUD placed, including women who received an IUD outside the allocated time window, those who had an IUD placed at surgery, and those who had IUD placed at clinics other than the study sites.

^c^Additional details regarding reasons for exclusion, discontinuation, and loss to follow-up within six months have been previously published ^8^.

^d^An additional five participants in the intervention group could not be reached at the 12-month follow-up. However, two of the 13 participants who did not respond at six months completed the 12-month questionnaire. Thus, the number of participants lost to follow-up between six and 12 months was three.

^e^An additional three participants in the control group could not be reached at the 12-month follow-up. However, one of the 11 participants who did not respond at six months completed the 12-month questionnaire. Hence, the number of participants lost to follow-up between six and 12 months was two.

^f^The denominator varies across analyses due to differences in data availability for specific outcomes (from questionnaires and/or medical records)
