# Supplementary material for: Immediate placement of intrauterine device after second‐trimester medical abortion—Secondary outcomes with one‐year follow‐up
Source: Acta Obstet Gynecol Scand. 2026 Jun 10;105(9):1672–9. doi: 10.1111/aogs.70259 (PMC13394263; doi:10.1111/aogs.70259)
Supplement: Supplementary file 3 [file AOGS-105-1672-s003.docx]

Supporting Information

Table S1. Poisson regression analysis of correlations between baseline covariates and IUD use at 12 months after second-trimester medical abortion (n=153^a^)

| Use of any type of IUD at 12 months after second-trimester abortion | | | |
| --- | --- | --- | --- |
| Baseline covariate | OR | 95% CI | *P*-value |
| Attended placement visit | 1.76 | 1.13 – 2.74 | 0.01 |
| Age below 30 years | 1.16 | 0.80 – 1.69 | 0.44 |
| Number of school years 12 or more | 0.99 | 0.73 – 1.34 | 0.94 |
| Parous | 0.93 | 0.62 – 1.39 | 0.73 |
| Previous abortion(s) | 1.05 | 0.76 – 1.44 | 0.77 |
| Positive health effects^b^ | 1.99 | 0.37 – 10.6 | 0.42 |
| Contraceptive effectiveness^b^ | 0.63 | 0.12 – 3.36 | 0.59 |
| Low risk of side-effects/low hormone dose^b^ | 1.74 | 0.32 – 9.45 | 0.52 |
| Recommendation from HCP^b^ | 0.57 | 0.11 – 3.06 | 0.51 |
| Comfortable/easy to use^b^ | 2.17 | 0.42 – 11.2 | 0.35 |

Abbreviations: IUD, intrauterine device; HCP, health care provider; OR, odds ratio; CI, confidence interval

^a^ A total of 15 participants withdrew their consent or were excluded, an additional 11 participants did not answer the question or chose multiple reasons as main reasons, these were also removed from the analysis.

^b^ Main reason reported for choosing a certain type of IUD.
