# Supplementary material for: Immediate placement of intrauterine device after second‐trimester medical abortion—Secondary outcomes with one‐year follow‐up
Source: Acta Obstet Gynecol Scand. 2026 Jun 10:10.1111/aogs.70259. Online ahead of print. doi: 10.1111/aogs.70259 (PMC13394263; doi:10.1111/aogs.70259)
Supplement: Supplementary file 3 — Table S2. Reasons for intrauterine device (IUD) discontinuation, other than expulsion, among women having an IUD placed after second‐trimester medical abortion (n = 127). [file AOGS-9999-0-s003.docx]

Table S2. Reasons for intrauterine device (IUD) discontinuation, other than expulsion, among women having an IUD placed after second-trimester medical abortion (n=127)

| Time after abortion | Within  3 months | Between 3-6 months | Between  6-12 months | Total during the first year after abortion |
| --- | --- | --- | --- | --- |
| Reasons |  |  |  |  |
| Bleeding problems | 3 | 3 |  | 6 |
| A wish to become pregnant | 2 | 2 | 1 | 5 |
| Abdominal pain | 1 | 1 |  | 2 |
| Recurrent vulvovaginal candidiasis and/or bacterial vaginosis |  |  | 2 | 2 |
| Infection | 1 | 1 |  | 2 |
| Other reason |  |  | 2^a^ | 2 |
| Mood change | 1 |  |  | 1 |
| Headache | 1 |  |  | 1 |
| Acne |  |  | 1 | 1 |
| No partner | 1 |  |  | 1 |
| Total | 20 | 8 | 6 | 34 |

^a^ Two participants reported discontinuation of IUD use due to other reasons; one accidentally removed the IUD when extracting a menstrual cup and the other participant reported “did not feel well, experienced several side effects”
